# Editorial: Over and under the skin: how our habits can influence cutaneous toxicity

**DOI:** 10.3389/ftox.2024.1476284

**Published:** 2024-08-20

**Authors:** Martina Iulini, Monday Ogaba Ogese

**Affiliations:** ^1^ Department of Pharmacological and Biomolecular Sciences, Faculty of Pharmacy, University of Milan, Milan, Italy; ^2^ Department of Hematology and Cellular Therapy, Cedars-Sinai Medical Center, Los Angeles, CA, United States

**Keywords:** skin, cutaneous toxicity, NAMs, health habits, skin sensitisation

## Introduction

As the largest organ of the human body, the skin is a critical barrier and interface between the internal and external environments. Skin health is constantly exposed to a variety of stimuli, including environmental chemicals and oral substances, and is therefore essential to overall health. The Research Topic “*Over and under the skin: how our habits can influence cutaneous toxicity*” in Frontiers in Toxicology examines the complex interactions between our habits and skin toxicity. This series of studies highlights the need to understand and mitigate skin reactions caused by modern lifestyles and ever-changing product use.

Based on the source mechanism, skin toxicity can be divided into contact dermatitis, photosensitivity, contact urticaria, chemical acne, pigmentary diseases, and drug- and tumor-related rashes. In addition, it can be classified according to the route of exposure, whether it is due to systemic effects or local skin irritation. In the past, many studies focused on analyzing skin toxicity caused by various substances. However, the continuous introduction of new pharmaceuticals, personal care products and other consumer products, as well as changes in habits and changes in the environment, require continuous research and new insights.

The aim of this Research Topic is to introduce an innovative approach to understand and control skin toxicity influenced by daily habits. So far, skin toxicity is often treated mainly through pharmacological and medical interventions, with less emphasis on lifestyle and habitual factors that contribute significantly to skin health. Typically, skin diseases are diagnosed based on visual inspection and patient-reported symptoms. Treatment is often based on topical applications or systemic medications to relieve symptoms. However, these approaches alone may not address the underlying habitual factors of skin toxicity, resulting in incomplete or temporary relief.

How can we improve the research, diagnosis and efficacy evaluation of skin toxicity treatments? How can we link daily habits to skin health? These questions are at the heart of this Research Topic, which explores the link between lifestyle and skin toxicity to propose comprehensive and sustainable solutions. By considering both external and internal factors, we can develop more effective prevention and treatment strategies.

Habitual factors such as diet, hygiene and exposure to environmental toxins play a vital role in skin health. However, these factors are often overlooked in favor of direct medical treatment. From dietary choices to skin care, a continuous stream of daily activities can affect the condition of the skin and its ability to respond to treatment. By focusing on these habits, we can better understand their cumulative impact on skin toxicity.

New technologies and methods allow us to precisely track and analyze habitual factors. For example, wearable devices can monitor UV exposure, skin moisture levels, and other relevant parameters, providing real-time data that can be used to customize personal skin care plans. This collection of articles proposes an integrated approach to treating skin toxicity and emphasizes the role of habits in influencing skin health. By considering both external and internal factors, we can develop more effective prevention and treatment strategies.

## Key contributions and themes

1. Standardization and international adoption of defined approaches for skin sensitization: this mini review written by Casati et al. discusses the efforts to integrate data from *in silico*, *in chemico*, and *in vitro* methods to ensure comprehensive mechanistic coverage of the skin sensitization Adverse Outcome Pathway (AOP). The development and international adoption of defined approaches for skin sensitization, as detailed in OECD guideline 497, represent a significant advancement. The guideline offers an alternative to animal testing by providing equivalent or superior information for regulatory purposes, setting a precedent for future regulatory acceptance of human biology-relevant new approach methodologies (NAMs).
2. Promoting NAMs for research on skin color changes in response to environmental stress factors: tobacco and air pollution. The review written by Bouchard and Costin explores how environmental stress factors, such as pollutants and tobacco smoke, impact the skin pigmentation. The complexity of these factors requires a thorough understanding of the mechanisms involved in skin hyperpigmentation. The study reported in the article highlights the role of reactive oxygen species and aryl hydrocarbon receptors in mediating these effects. NAMs are emphasized for their ability to provide mechanistic insights, aiding in the design of ingredients and formulations to counter these impacts.
3. Why drug exposure is frequently associated with T-cell mediated cutaneous hypersensitivity reactions: cutaneous hypersensitivity reactions are a common manifestation of drug allergies, ranging from mild rashes to severe conditions like Stevens Johnson syndrome. This review written by Line et al. addresses the cellular and metabolic mechanisms driving these reactions, particularly the role of tissue-specific antigen-presenting cells and reactive drug metabolites. The article underscores the skin’s unique immune environment and its propensity for hypersensitivity reactions, providing a comprehensive overview of current research and future directions.
4. Development of skin diseases following systemic exposure: example of dioxins: dioxins, particularly 2,3,7,8-tetrachlorodibenzo-p-dioxin (TCDD), can cause skin diseases following systemic exposure. This mini review written by Sorg and Saurat examines the mechanism by which TCDD induces skin hamartomas, historically known as chloracne. The findings highlight the disruption of sebocyte differentiation and migration, leading to skin lesions. Understanding these mechanisms is crucial for developing preventive and therapeutic strategies for dioxin-induced skin conditions.
5. Case report: envafolimab causes local skin necrosis: Liu et al. present a case study of envafolimab, a PD-L1 inhibitor administered subcutaneously, associated with severe skin necrosis in a patient with hepatocellular carcinoma. This case emphasizes the need for thorough post-marketing surveillance and additional clinical studies to ensure the safety and efficacy of new drugs. The article discusses potential immune mechanisms leading to local skin necrosis and highlights considerations for the clinical use of PD-L1 inhibitors.
6. Bioactivation of cinnamic alcohol in a reconstructed human epidermis model and evaluation of sensitizing potency of the identified metabolites: Ndreu et al. investigate the bioactivation of cinnamic alcohol, commonly used in fragrances, using a reconstructed human epidermis model. Two metabolites, pOH-cinnamic alcohol and pOH-cinnamic aldehyde, were identified, with the latter being a moderate sensitizer. The research underscores the importance of identifying sensitizing metabolites for effective patch testing and improving safety assessments of skin products.

## Broader context and future directions

The findings within this Research Topic not only advance our understanding of cutaneous toxicity but also underscore the broader implications for public health and regulatory practices. As new substances continue to enter the market, the dynamic interplay between our habits and skin health necessitates ongoing research and vigilant regulatory oversight. The integration of NAMs in toxicity testing represents a promising advancement, ensuring that new products are both effective and safe for consumers.

In conclusion, the Research Topic “*Over and under the skin: how our habits can influence cutaneous toxicity*” offers valuable insights into the complex relationship between modern lifestyles and skin health. By addressing the toxicological impacts of various substances and promoting the use of innovative testing methodologies, this collection of studies paves the way for safer consumer products and improved public health outcomes.

For more detailed insights, For more detailed insights, explore the individual articles within this Research Topic.

